# Navigating collateral sensitivity: insights into the mechanisms and applications of antibiotic resistance trade-offs

**DOI:** 10.3389/fmicb.2024.1478789

**Published:** 2024-10-24

**Authors:** Hafij Al Mahmud, Catherine A. Wakeman

**Affiliations:** Department of Biological Sciences, Texas Tech University, Lubbock, TX, United States

**Keywords:** collateral sensitivity, antibiotic resistance trade-off, antibiotic therapy, CS mechanism, clinical implication

## Abstract

The swift rise of antibiotic resistance, coupled with limited new antibiotic discovery, presents a significant hurdle to global public health, demanding innovative therapeutic solutions. Recently, collateral sensitivity (CS), the phenomenon in which resistance to one antibiotic increases vulnerability to another, has come to light as a potential path forward in this attempt. Targeting either unidirectional or reciprocal CS holds promise for constraining the emergence of drug resistance and notably enhancing treatment outcomes. Typically, the alteration of bacterial physiology, such as bacterial membrane potential, expression of efflux pumps, cell wall structures, and endogenous enzymatic actions, are involved in evolved collateral sensitivity. In this review, we present a thorough overview of CS in antibiotic therapy, including its definition, importance, and underlying mechanisms. We describe how CS can be exploited to prevent the emergence of resistance and enhance the results of treatment, but we also discuss the challenges and restrictions that come with implementing this practice. Our review underscores the importance of continued exploration of CS mechanisms in the broad spectrum and clinical validation of therapeutic approaches, offering insights into its role as a valuable tool in combating antibiotic resistance.

## Introduction

As pathogens replicate at sites of infection, mutational events can occur that lead these cells down distinct evolutionary trajectories. One of the most clinically concerning evolutionary adaptations is the acquisition of resistance mechanisms to various antimicrobials that were initially crafted to eliminate or hinder microbial growth. The swift development of bacterial resistance is linked to the extensive and improper use of antibiotics (Aslam et al., 2018). For instance, the suggested treatments to suppress chronic conditions in cystic fibrosis (*CF*) patients involve continuous antibiotic exposure, increasing the likelihood of the emergence of multidrug- or extensive drug resistance (Kavanaugh et al., 2021). Conversely, the identification of new antimicrobial agents falls significantly behind the rate at which resistance is evolving in pathogenic microbes (Jackson et al., 2018). The decline in antimicrobial drug discovery rates is attributed to heightened risk, low profitability, and changing priorities in the pharmaceutical industry (Power, 2006). Hence, the rise of resistance, coupled with the insufficient progress in developing new drugs, has significantly diminished our capacity to effectively combat bacterial infections. Thus, there is a rising interest in using current antibiotics to devise treatment approaches that not only eradicate undesirable bacteria or pathogenic bacteria but also prolong the effectiveness of existing antibiotics.

The development of antimicrobial resistance arises from genetic changes impacting various physiological processes within bacteria that may ultimately alter a particular trait. A trade-off arises when enhancing fitness through one component mechanism/trait unavoidably leads to a decline in fitness/functioning through another component mechanism/trait, thereby imposing a ceiling on the overall attainable fitness/function (Cohen et al., 2020). This phenomenon is attributed to pleiotropic mutations that influence multiple characteristics (Ardell and Kryazhimskiy, 2021). These genetic alterations due to random mutation in the bacterial genome can provide them a particular advantage at the cost of other disadvantages, and this potential trade-off can be seen between different traits or characteristics in bacteria (Ferenci, 2016). The development of resistance to a particular antimicrobial agent may result in collateral effects, modifying responses to other drugs, with important implications for antibiotic treatments and susceptibility across environments (Ardell and Kryazhimskiy, 2021; Sørum et al., 2022). Recognizing strong patterns in the evolution of antibiotic resistance and associated resistance trade-offs, like collateral sensitivity (CS), is essential for developing rational treatment approaches (Sanz-García et al., 2023). Diverse resistance mutations can result in contrasting collateral effects; some may heighten the bacteria’s sensitivity (collateral sensitivity) to a second drug, while others could increase resistance to other treatments (collateral resistance; CR) (Ardell and Kryazhimskiy, 2021). In addition to bacterial species, cancer cell lines have shown that collateral sensitivity or resistance can extend to other drugs that are structurally or functionally unrelated (Waller et al., 2023). While clinical cases have reported bacterial resistance to phages, various investigations into the trade-offs in fitness between antibiotic and phage resistance have opened up potential possibilities in the field of phage therapy alongside antibiotic resistance-based CS therapy (Fujiki et al., 2023). In this review, our focus mainly lies on CS in bacteria that represents an evolutionary trade-off involving various resistance mechanisms (Ma et al., 2023). Genetic analyses are commonly employed in research studies to delve into the molecular mechanisms of CS in microorganisms (Ma et al., 2023; Lázár et al., 2013).

The potential of CS in treatment design could be employed to restrict the reemergence of infection by suppressing or even reversing resistance evolution (Ma et al., 2023). Such treatment design may include antibiotic combination therapy, which has proven effective and has been used for decades to combat the spread of various clinically significant infectious diseases such as HIV and tuberculosis (Seo et al., 2022; Mahmud et al., 2021; Gilliam et al., 2006). Combination therapy is capable of broadening the bacterial target spectrum to encompass resistant variants, achieving synergistic therapeutic outcomes, and potentially preventing the emergence of resistance (Xu et al., 2022). The efficacy of such multi-drug combination treatments depends on the ability to predict whether mutations conferring resistance to one drug also render the bacteria sensitive to an alternative. Employing two or more existing drugs sequentially or through cycling can be a viable approach to restrict the emergence of drug-resistant pathogens and ultimately eliminate resistant bacteria (Kavanaugh et al., 2021; Ardell and Kryazhimskiy, 2021). In addition to swiftly eradicating drug-resistant variants, a multi-drug treatment approach based on CS may also reduce treatment times (Waller et al., 2023; Imamovic and Sommer, 2013). Overall, this combination treatment based on CS may manifest three scenarios. First, bacteria would often fail to withstand hypersensitivity and become extinct. Second, hypersensitivity would occasionally transform bacteria into multidrug resistance. Third, the acquisition of resistance would frequently lead to re-sensitization to the other drug (Barbosa et al., 2019).

This review outlines the basics of antibiotic resistance acquisition, explores how antibiotic resistance in bacteria may render cells susceptible to other antibiotics through trade-offs, delves into numerous reported cases of collateral sensitivity and resistance in clinically important pathogens with their respective molecular mechanisms, and discusses the challenges and opportunities for collateral sensitivity-based treatment along with its clinical implications. We contend that studying CS holds promise in addressing the resistance crisis and treating pathogens adapting to antibiotics and has the potential to restrict the emergence of antibiotic resistance (Yekani et al., 2023).

## Antibiotic resistance

### Evolution of antibiotic resistance

Bacteria may endure bactericidal antibiotic treatment in three ways: persistence, tolerance, and resistance. Bacteria may develop a transient persister phenotype when a small part of the whole population survives against a prolonged treatment with a high dose of antibiotics. Following treatment, unlike resistant bacteria, most of these persister cells become re-sensitized to that particular antibiotic (Cabral et al., 2018; Fisher et al., 2017). Persister cells usually reduce antibiotic uptake and target availability by downregulating their essential metabolism, which reduces their susceptibility to antibiotic therapy (Cabral et al., 2018; De Groote et al., 2009). Antibiotic persistence and tolerance are frequently used interchangeably, but the distinction between these two terms remains unclear (Brauner et al., 2016). Yet, according to many scientists, persistence specifically applies to a bacterial subpopulation, whereas tolerance relates to the ability of the entire bacterial population to endure antibiotic exposure (Brauner et al., 2016; Huemer et al., 2020; Michaux et al., 2022). Tolerance conferred by environmental factors such as growth conditions that impact cellular metabolism enables bacterial cells to survive a transient exposure to antibiotics at concentrations that would otherwise be lethal (Brauner et al., 2016; Kester and Fortune, 2014). On the other hand, antibiotic-resistant bacteria are easily identified by their ability to thrive in the drug’s presence due to their heritable genomic alteration (Balaban et al., 2019; Gollan et al., 2019). Antibiotic resistance can be achieved through either random mutation on the chromosome, known as spontaneous mutation, or horizontal gene transfer (HGT) involving acquisition of foreign genetic material, such as plasmids (Davies and Davies, 2010; Rosenkilde et al., 2019; Neu, 1992). The process of bacterial evolution, driven by genetic mutations over time, is a major contributor to antibiotic resistance, making disease-causing pathogens resistant to multiple drugs and impervious to antibiotic treatment (Barbosa et al., 2019). Studies have demonstrated that resistance can evolve even in the absence of antibiotics. For example, extended iron starvation may select *Staphylococcus aureus* for mutations that confer aminoglycoside resistance (Islam et al., 2022) and selective exposure to antibiotics can effectively enrich these resistant organisms (Sanz-García et al., 2023). The widespread misuse of antimicrobials in clinical and agricultural settings has led to significant resistance evolution through selective pressure, becoming a major cause of antibiotic resistance in bacteria over the past few decades (Marston et al., 2016; Hasan et al., 2023; Alumran et al., 2012).

### Mechanisms of antibiotic resistance

The primary antibiotic resistance mechanisms include inactivating drugs, restricting drug uptake, actively expelling drugs, and modifying drug targets (Reygaert, 2018). Antibiotic effectiveness may be compromised by bacterial pathogens that produce resistance enzymes. These enzymes can either hydrolyze or chemically modify the antibiotics, rendering them ineffective against microorganisms. A common resistance mechanism in pathogenic bacteria against aminoglycosides, *β*-lactams, and chloramphenicol is the enzymatic inactivation of antibiotics, either by hydrolyzing them or modifying them into inactive derivatives (Davies, 1994). A class of inactivating enzymes known as aminoglycoside-modifying enzymes (AMEs) catalyzes the addition of chemical groups to particular residues of aminoglycoside molecules, changing the drug’s structure and lowering its affinity for attaching to its target. AMEs are classified according to the type of group they transfer, such as acetyltransferases (AACs), nucleotidyltransferases (ANTs), and phosphotransferases (APHs), as well as the specific residue they modify and the resistance profile they provide (Pradier and Bedhomme, 2023). Additionally, *β*-lactamases break down the β-lactam ring, which disrupts the drug’s ability to interact with its target, rendering it ineffective in killing pathogens (Costa et al., 2020). Another antibiotic that is frequently made inactive by different enzymes is chloramphenicol. The most frequent inactivation is caused by the hydroxyl group at the C3 position being acetylated by the enzyme chloramphenicol acetyltransferase (CAT). Furthermore, phosphotransferase can inactivate chloramphenicol by O-phosphorylation. Similarly, chloramphenicol acetate esterase gene (estDL136)-containing *Escherichia coli* cell extracts has also been shown to hydrolyze chloramphenicol (Tao et al., 2012).

Restricting the uptake of antibiotics is another way that microbes evade antibiotic therapy. Porins in the outer membrane allow drugs such as fluoroquinolones, chloramphenicol, and β-lactams to enter Gram-negative bacteria. Thus, changes in these porins’ quantity, size, or selectivity may have an impact on the rate at which these antibiotics permeate into cells (Kumar and Schweizer, 2005). A clinical isolate of *Serratia marcescens* that was resistant to both aminoglycosides and β-lactams was one of the first cases of antibiotic resistance linked to porin depletion that was reported (Kumar and Schweizer, 2005).

Bacteria can develop resistance by increasing their natural efflux activity, which may occur due to overexpression or mutations in the genes that control energy-dependent transporters. When faced with antibiotics or toxins, efflux is often the quickest and most effective mechanism for bacteria to resist stress (Du et al., 2018). There are five fundamental groups of efflux pumps: SMR (small multidrug resistance), MFS (major facilitator superfamily), MATE (multi antimicrobial extrusion), RND (resistance nodulation and cell division), and ABC (ATP binding cassette). Other than ABC, the remaining four families of transporters rely on a proton gradient as an energy source, while transporters in the ABC family use energy originated from ATP hydrolysis (Pucelik and Dąbrowski, 2022). Multidrug efflux pumps may reduce intracellular antibiotic levels and block the drug from getting into its target within the cell. Additionally, these pumps typically have the capacity to expel a wide range of antimicrobial substances outside the bacterium, leading to commonly observed intrinsic, transient, inducible, or acquired antimicrobial resistance (Gil-Gil et al., 2023; Hernando-Amado et al., 2016).

Various other forms of antibiotic resistance can emerge, with enzymatic alterations of drug targets becoming a significant concern in clinical settings. For example, changes to the pentapeptide stems in peptidoglycan precursors lead to glycopeptide resistance, and modifications in lipopolysaccharides are associated with polymyxin resistance. Furthermore, resistance to many ribosome-targeting antibiotics is conferred by methylation of the ribosome (Schaenzer and Wright, 2020). Target site alterations usually result from spontaneous mutations in the bacterial chromosomal genes, which are subsequently selectively favored when antibiotics are present. For example, resistance to rifamycins can be acquired by mutations in RNA polymerase, but resistance to quinolones can be acquired through modifications in DNA gyrase (Lambert, 2005). In some instances, bacteria may alter antibiotic targets through the transfer of resistance genes from other organisms via various methods of genetic exchange. For example, horizontal gene transfer plays a vital role in the spread of the *mecA* gene among the *S. aureus* population, with this gene being responsible for high-level methicillin resistance by encoding an alternative penicillin-binding protein referred to as PBP2a (Lambert, 2005; Wielders et al., 2002). In addition, glycopeptide antibiotics, like teicoplanin and vancomycin, attach to the d-Ala-d-Ala termini of peptidoglycan precursors to prevent the production of cell walls. The *vanA* and *vanB* gene clusters are primarily responsible for resistance, as they produce d-Ala–d-Lac precursors with a lower affinity for these antibiotics (Arthur and Quintiliani Jr, 2001). The spread of vancomycin-resistant enterococci (VRE) in global healthcare may driven by clonal transmission and horizontal transfer of these two genes *vanA* and *vanB*, particularly via membrane vesicles (Lehmkuhl et al., 2024).

### Clinical implications of antibiotic resistance

Shortly after the first antibiotic penicillin entered clinical use, the enzyme penicillinase, which neutralized its effectiveness, was discovered (Virk and Steckelberg, 2000). The overuse or misuse of antibiotics, prolonged hospital stays, an increase in the number of immunocompromised patients, and the frequent use of invasive procedures and devices are some of the reasons contributing to the rise in antimicrobial resistance (Virk and Steckelberg, 2000). It is concerning that diverse clinical strains are evolving to become pan-resistant, indicating resistance to all available antibiotics (Nikaido, 2009). The most extensive study to date revealed approximately 4.95 million deaths globally in 2019 linked to bacterial antimicrobial resistance (AMR), with projections indicating a potential increase to 10 million annual deaths by 2050 (Murray et al., 2022; O’Neill, 2016). Multidrug-resistant pathogenic strains are a significant threat in treatment and may cause increased mortality and morbidity, mainly in immune-compromised vulnerable patients (Haney and Hancock, 2022; Azimi and Rastegar Lari, 2017). The inability to completely eradicate infections, the need for more invasive procedures to get rid of deeply ingrained illnesses, and the increased risk of recurrence are all associated with greater rates of morbidity and mortality (Virk and Steckelberg, 2000). In addition to higher mortality and morbidity rates, antimicrobial resistance can lead to prolonged hospital stays, and increased treatment costs (Maragakis et al., 2008). The cost for patients with infections from antimicrobial-resistant organisms is significantly higher, typically ranging between six thousand and thirty thousand US dollars, than for those infected by antimicrobial-susceptible organisms. This gap in expenses becomes even more significant when comparing individuals with resistant infections to those who are not infected at all (Maragakis et al., 2008; Cosgrove, 2006). Additionally, when patients are infected with antimicrobial-resistant pathogens, delaying the proper antimicrobial treatment can have detrimental effects (Maragakis et al., 2008).

Long-term and indirect impacts of resistance on patients can be equally as severe as the short-term direct effects, which are captured by mortality rates and lengths of hospital stays. For instance, even if MRSA (methicillin resistant *S. aureus*) is not the current cause of the infection, a patient with a history of MRSA infections is usually isolated and given vancomycin treatment when they experience a fever (Eliopoulos et al., 2003). Antimicrobial resistance affects even patients without resistant infections, as rising resistance levels in common pathogens necessitate the use of broad-spectrum antibiotics for empirical treatment. These medications are often more expensive, can harm protective microflora, and may be more toxic or less effective (Eliopoulos et al., 2003). Hospitals and other healthcare providers could be more inclined to support measures like infection-control programs if they are aware of how resistance affects clinical and financial results (Perencevich et al., 2007).

## Trade-off in antibiotic resistance

### Definition and concept of collateral sensitivity

Trade-offs, observed in various aspects of our lives, including engineering, economy, and nature, involve a balance where beneficial changes in one trait are typically associated with detrimental changes in other traits, as supported by a wealth of comparative and experimental data (Bennett and Lenski, 2007; Velicer et al., 1999). Similarly, trade-offs are prevalent in the microbial world, where tolerant populations, while displaying enhanced survival under bactericidal drug treatment, incur the cost of impaired proliferation during infection (Michaux et al., 2022). Additionally, trade-offs are evident in antibiotic sensitivity, as the development of resistance to one antibiotic can result in heightened sensitivity to other unrelated antibiotics, referred to as CS (Barbosa et al., 2019; Pál et al., 2015; Aulin et al., 2021; Baym et al., 2016; Li et al., 2022; Hernando-Amado et al., 2023a). This phenomenon holds the potential to restrict the emergence of antimicrobial resistance (Aulin et al., 2021). In short, resistance mutations can interfere with cellular functions, efflux pump systems, or metabolic pathways, leading to a series of downstream effects that increase the susceptibility of the bacterium to a different antibiotic (Hernando-Amado et al., 2023a).

### Importance of collateral effects on treatment

While the idea of CS was initially described by Bryson and Szybalski in the 1950s, who observed increased sensitivity to polymyxin B in an *Escherichia coli* strain after acquiring chloramphenicol resistance, it has now become a topic of significant interest among researchers (Szybalski and Bryson, 1952). For example, many studies have explored CS in various clinically pathogenic microbes, including *E. coli* (Barbosa et al., 2019; Munck et al., 2014; Podnecky et al., 2018; Liu et al., 2023), *Staphylococcus aureus* (Gonzales et al., 2015), *Pseudomonas aeruginosa* (Barbosa et al., 2019; Imamovic et al., 2018), *Salmonella Typhimurium* (Hasan et al., 2023), *Burkholderia multivorans* (Kavanaugh et al., 2021), *Klebsiella pneumoniae* (Ma et al., 2023; Hobson et al., 2022), *Salmonella enterica* (Malik et al., 2017), etc. to design rational therapies and to restrict resistance reemergence.

Contrary to the negative interactions among antibiotics represented by CS, positive evolutionary interactions, termed collateral/cross resistance (CR), can lead to enhanced resistance against another antibiotic (Pál et al., 2015). This occurrence is a result of acquired resistance mechanisms, including genetic mutations at the mutual target site, elevated activity of efflux pumps, and decreased antibiotic uptake. These mechanisms provide resistance to a particular antibiotic and can extend resistance to other antibiotics sharing similar structures, target sites, or functions (Kaviya et al., 2023). The evolutionary CR patterns to clinical antibiotics are attracting intensive attention due to potential fitness costs and trade-offs that shape the evolutionary trajectory on rugged fitness landscapes (Theuretzbacher et al., 2020; Nichol et al., 2019). Both CS and CR have been reported in different research. For example, adapted ciprofloxacin resistance in *P. aeruginosa* may make them sensitive to tobramycin and piperacillin (Yen and Papin, 2017). The development of aminoglycoside resistance has been documented to result in heightened susceptibility to various antibiotic classes, including *β*-lactams, chloramphenicol, fluoroquinolones, tetracyclines, and doxycycline (Ma et al., 2023; Lázár et al., 2013). Similarly, spontaneous mutation may introduce CR to different antibiotics; for example, *S. typhimurium* adapted to gentamicin may become resistant to kanamycin, and additionally, kanamycin-adapted *S. typhimurium* may also become resistant to gentamicin (Hasan et al., 2023). The significant consequences of CR in pathogenic microorganisms becomes evident as resistance to a particular antibiotic leads to evolved resistance to other antibiotics within the same or different classes, severely limiting therapeutic options and rendering antibiotics ineffective in treating pathogenic microbes (Sincak et al., 2023; Dawan and Ahn, 2020). Beyond bacterial resistance, phage have also played a substantial role in influencing the evolution of bacterial communities and populations, driven by a co-evolutionary mechanism referred to as an arms race (Sørum et al., 2022). Specifically, the development of phage resistance might have the potential to reinstate bacterial sensitivity to antibiotics (Oromí-Bosch et al., 2023; Mangalea and Duerkop, 2020).

### Employing collateral sensitivity in antibiotic therapy

Antibiotic resistance poses a significant global public health challenge, and the slow discovery of new antibiotics necessitates novel strategies for effective bacterial infection treatment. While the exploration and exploitation of combination therapy based on physiological interactions, such as synergy and antagonism, have been ongoing for decades, attention to evolutionary interactions leading to collateral effects has only recently begun (Mahmud et al., 2021; Pál et al., 2015). For instance, in addressing infectious diseases such as HIV, malaria, and tuberculosis, the conventional practice involves utilizing combination therapy as the standard approach (Gogtay et al., 2013). In the context of CS-based combination therapy, particular drug combinations are anticipated to postpone the development of resistance (Pál et al., 2015). In principle, the heightened sensitivity observed during the administration of the second antibiotic could potentially result in a more effective eradication of the bacterial population. This could occur by diminishing the pool of surviving cells, thereby limiting the emergence of resistant mutants, or by rendering the impact of resistance mutations inadequate for their widespread dissemination (Brepoels et al., 2022). The success of optimal antibiotic combinations is determined by the interplay of physiological drug interactions (such as synergism or antagonism) and the prevalence of mutations with pleiotropic fitness effects, which encompass cross-resistance/collateral resistance and cross-sensitivity/collateral sensitivity (Lázár et al., 2013; Rosenkilde et al., 2019; Pál et al., 2015; Chait et al., 2007). Comprehensive systematic recognition of CR holds clinical significance, aiding in the avoidance of empirically unfavorable antibiotic combinations during treatment. On the other hand, CS can be leveraged for the development of strategies in antibiotic combination treatments aimed at suppressing the emergence of resistance (Imamovic et al., 2018; Rosenkilde et al., 2019; Yeh et al., 2009; Amsalu et al., 2020).

Results from experimental studies have been encouraging, indicating high rates of bacterial extinction and low occurrences of (multi) drug resistance evolution with certain drug combinations. Moreover, there is a noticeable correlation between the mutational patterns associated with resistance and the types and order of drugs used (Brepoels et al., 2022). CS-based treatment strategies offer flexibility in combining antibiotics with a CS relationship, such as simultaneous administration, sequential administration, or cyclic (alternating) administration (Aulin et al., 2021). In determining the appropriate treatment strategy, the initial step involves observing either a unidirectional or reciprocal relationship in terms of collateral sensitivity within drug pairs. The latter, characterized by mutual sensitivity, is particularly well-suited for designing strategies aimed at suppressing resistance through cycling but is not necessarily essential to suppress antibiotic resistance (Aulin et al., 2021; Zwep et al., 2021). Achieving optimal results in sequential therapy through the exploitation of evolved CS relies on considerations such as the specific order and combination of drugs, the extent of CS effects, broader adaptation trade-offs, and the presence of epistatic genetic interactions (Barbosa et al., 2019). The effectiveness of cycling therapies based on CS is also heavily contingent on the order in which drugs are administered (Aulin et al., 2021).

## Potential cases and mechanisms of collateral sensitivity

In recent days, the implications of collateral effects are widespread, yet their anticipation proves challenging due to the inherent variability in the response of distinct populations exposed to the same drug. These independent groups may manifest distinct profiles of CS and demonstrate significantly divergent levels of fitness costs (Maltas and Wood, 2019). Various researchers have utilized diverse methods to delve into the potential mechanisms of action for different CS and CR. One prevalent approach involves the application of whole-genome sequence analysis, wherein the evolved sequences are compared with those of the parental strains (Lázár et al., 2013; Lázár et al., 2014). This method offers insights into the genetic changes that may underlie the observed CS or CR, providing a comprehensive understanding of the mechanisms at play. We have explored certain reported instances of potential CS and CR along with their corresponding mechanisms of action in relation to the respective pathogens, which are categorized by pathogen below and summarized in Table 1.

**Table 1:** Potential collateral sensitivity (CS) cases, their mechanisms, and molecular basis.

Strains	CS; Uni-directional (R > S) bi-directional (R<>S)	Altered physiology	Gene mutation	CR (gene mutation)	Ref.
*Acinetobacter baumannii*	TIG > SUL	Two-component system sensor	*adeS*		Yang et al. (2023)
	COL > VAN, TOB	Synthesis inhibition of Lipid A domain of Lipopolysaccharide/lipooligosaccharide (LPS/LOS)			Boll et al. (2016)
*Bacillus subtilis*	VAN > BLA	Altered cell surface	*ykcB*		Ishikawa et al. (2022)
*Burkholderia multivorans*	ß-LAC, MER > LEV, MIN, CHL, SXT	Altered cell wall			Flanagan et al. (2020)
	SXT > MER, CEFT, LEV, MIN, CHL	RND efflux pump regulators or other pumps			Kavanaugh et al. (2021)
	MER <> SXT, MER <> LEV, CEFT <> SXT, MER <> MIN, CEFT <> LEV		(LEV > MER*; rseP, dacB*, and *fimV*) (MER > LEV*; rimO, snoaL-*like polyketide cyclase*, rhaT*) (LEV > CEFT*; bpeR, fimV*, phage lysozyme) (CEFT > LEV*; rne, tRNA-ser, dacB, fabI*, and a transmembrane protein)		Kavanaugh et al. (2020)
*Enterococcus faecalis*	AMPI, OXA, CEFTR, FOS > RIF	Cell wall synthesis inhibition			Maltas and Wood (2019)
	DAP > CEFTR	LiaFSR two-component system	*liaX*	DAP > CEFTR (*cls, bceR, ycvR*)	Huynh et al. (2023)
*Enterococcus faecium*	VAN > LEF	ABC-F protein that shields bacterial ribosomes from antibiotic targeting	*van* gene cluster, *msrC*		Li et al. (2022)
*Escherichia coli*	AMG > BLA	Two regulatory systems (PhoP-PhoQ and PmrBPmrA)	*pmrB*		Lázár et al. (2013) and Baym et al. (2016)
	MEC, PRO, TIG > NIT	Upregulation of nitroreductase activity, resulting in overexpression of oxygen-insensitive nitroreductases NfsA and/or NfsB	*hemL* and *lon, spoT*		Roemhild et al. (2020)
	AMG > BLA, FLU, CHL, TET, DOX	Decrease AcrAB-TolC activity Diminished membrane potential	*TrkH, CyoB, IspA* and *HemA*		Lázár et al. (2013)
	MEC, PIP-TAZ > CEFO		*bla_CTX-M-15_*		Rosenkilde et al. (2019)
	CAR > AZI, COL	Harboring plasmid pOXA-48			Herencias et al. (2021)
	AMPI, CEFOX, TET > GLA, HBD3	Induction of the AcrAB-TolC Increases the expression of the kinase WaaY	*marA*		Lázár et al. (2018)
	CEP > BOR	Altered cell wall biosynthesis	tRNA ligase (ThrRS)		Liu et al. (2023)
*Klebsiella pneumoniae*	TET, AMG <> CAR	Membrane potential Oxidiation-reduction process SoxRS-MarAB-AcrAB			Zhao and Drlica (2014)
	CAR > BLA-BLAI, (CEFT-AVI), (AZT-AVI) combination	Overexpression of the porin proteins OmpA and OmpU		CAR > ERA	Xu et al. (2022)
	(CEFT-AVI) combination > IMI, MER, ERT	Decreased carbapenemase			Hobson et al. (2022)
*Mycobacterium tuberculosis*	ISO, RIF, AMI, STR, LEV, OFL, ETH, ETHI, PYR > BLA	Activation of the key inhibitor of β-lactam resistance	*blaI*		Trigos et al. (2021)
		Upregulation of efflux pump MmpL15 (CR)	*rv0678*	BED > CLO	Hartkoorn et al. (2014)
	INH > BED, TB47, PA824, TAC, Q203		*katG*		Waller et al. (2023)
	RIF, FIX, BDQ, INH > PA824		*rpoB* (i.e., RIF and FIX)*, atpE* (i.e., BED)*, and katG* (i.e., INH)		Waller et al. (2023)
*Pseudomonas aeruginosa*	CIP > IMI, PIP, TOB, β-LAC, CEFT, CEFE, CEFO, PIP-TAZ, AZT, MER	Overexpression of MexCD-OprJ MexAB-OprM, MexXY-OprM AmpC-lactamase	*nfxB*		Mulet et al. (2011)
	CIP, AZI > COL, AMG	Efflux pump	*nfxB*		Imamovic et al. (2018)
	BLA > AMG		*nalC or mexZ*		Barbosa et al. (2017)
	GEN > PEN	Two-component regulatory system	*pmrB*		Barbosa et al. (2017)
	CEFT > TOB	Resistance efflux pump	*mexXY*		Hernando-Amado et al. (2020)
	TOB, CEFT, TIG > FOS	Disruption in peptidoglycan-recycling	*fosA*		Laborda et al. (2022) and Genova et al. (2023)
	CIP > TOB	Overexpression of MexCD-OprJ	*nfxB*		Hernando-Amado et al. (2023a)
	CIP > TOB, AZT				Hernando-Amado et al. (2023b)
	GEN > PEN	Two-component regulatory system	*pmrB*		Jansen et al. (2016)
	BLA > AMG		*nalC or mexZ*		Jansen et al. (2016)
*Salmonella enterica*	COL, PRO > CYCO2	Lipopolysaccharide biosynthesis	*pmrA* (COL) *hemL* (PRO)		Malik et al. (2017)
*Staphylococcus aureus*	SXT > MER, CEFT, LEV, MIN, CHL	RND efflux pump regulators or other pumps			Kavanaugh et al. (2021)
	VAN, DAP > BLA; VAN, DAP, TEI > CFRL				Barber et al. (2014)
	DAP > BLA	Altered posttranscriptional maturation of penicillin binding protein 2a	*mprF*		Renzoni et al. (2017)
	DAP, VAN, DAL > BLA	Increased phosphatidylglycerols composition on membrane		DAP, VAN, DAL > GLY, LIP, LIPG	Hines et al. (2020)
	DAP > OXA				Yang et al. (2010)
	VAN > CEFR				Werth et al. (2013)
*Staphylococcus haemolyticus*	DAP > PEN, CEFOX				Vignaroli et al. (2011)
*Salmonella typhimurium*	MER <> SXT, MER <> LEV, CEFT <> SXT, MER <> MIN, CEFT <> LEV		(LEV > MER*; rseP, dacB*, and *fimV*) (MER > LEV*; rimO, snoaL-*like polyketide cyclase*, rhaT*) (LEV > CEFT*; bpeR, fimV*, phage lysozyme) (CEFT > LEV*; rne, tRNA-ser, dacB, fabI*, and a transmembrane protein)		Kavanaugh et al. (2020)
Phage	Phage > antibiotic, antimicrobial peptide	Altered polysaccharides on bacterial membrane			Fujiki et al. (2023)
	Phage > antibiotic	Altered efflux pump			Fujiki et al. (2023)
	Phage > serum immune components, COL	Structural alterations in lipopolysaccharide			Ruest et al. (2023)

AMG, Aminoglycoside; AMI, Amikacin; AMP, Antimicrobial peptide; AMPI, Ampicillin; AVI, Avibactam; AZI, Azithromycin; AZT, Aztreonam; BED, Bedaquiline; BOR, Borrelidin A; CAR, Carbapenem; CEFE, Cefepime; CEFO, Cefotaxime; CEFOX, Cefoxitin; CEFOZ, Cefozopran; CEFP, Cefpirome; CEFR, Ceftaroline; CEFT, Ceftazidime; CEFTR, Ceftriaxone; CEP, Cephalosporin; CEP, Cephems; CHL, Chloramphenicol; CIP, Ciprofloxacin; CLO, Clofazimine; COL, Colistin; CYCO2, Cyclotide Cycloviolacin O2; DAL, Dalbavancin; DOX, Doxycyline; ERA, Eravacycline; ERT, Ertapenem; ETH, Ethambutol; ETHI, Ethionamide; FIX Fidaxomicin; FLU, Fluoroquinolones; FOS, Fosfomycin; GEN, Gentamycin; GLA, Glycine-leucine-amide; GLY, Glycopeptides; HBD3, Human beta defensin-3; IMI, Imipenem; ISO, Isoniazid; KAN, Kanamycin; LEF, Lefamulin; LEV, Levofloxacin; LIP, Lipopeptides; LIPG, Lipoglycopeptides; MEC, Mecillinam; MER, Meropenem; MIN, Minocycline; NIT, Nitrofurantoin; OFL, Ofloxacin; OXA, Oxacillin; PEN, Penicillin; PIP, Piperacillin; PIP-TAZ, Piperacillin-Tazobactam; PRO, Protamine; PYR, Pyrazinamide; RIF, Rifampicin; STR, Streptomycin; STR, Streptomycin; SUL, Sulbactam; SXT, Trimethoprim-sulfamethoxazole; TAZ, Tazobactam; TET, Tetracycline; TIG, Tigecycline; TIG, Tigecycline; TOB, Tobramycin; VAN, Vancomycin; BLA, β-lactams. CS, collateral sensitivity; CR, collateral resistance; R, resistance; S, sensitivity.

### Acinetobacter baumannii

Four XDR *A. baumannii* strains were sequentially isolated from a single bacteremia patient in order to investigate the in-host evolution and CS phenomenon. This demonstrated a shift in the patient’s susceptibility to antibiotics. The altered susceptibility phenotype was shown to be attributed to the chromosomal multiplication of blaOXA-23 inside Tn2006, whereas the appearance of CS to sulbactam in tigecycline adapted strain was associated with a mutation of the two-component system sensor *adeS* (Yang et al., 2023).

Lipopolysaccharide/lipooligosaccharide (LPS/LOS) is a major factor in antibiotic resistance, and the outer membrane of gram-negative bacteria acts as a blockade against toxins and antibiotics. Colistin, a last resort for resistant infection, targets the synthesis of lipid A domain of LPS/LOS to damage bacterial cells. However, *A. baumannii* may evolve to resist colistin by not synthesizing lipid A on the membrane. Remarkably, the susceptibility of *A. baumannii* to vancomycin and tobramycin was enhanced upon the removal of lipid A/LOS from the outer membrane (Boll et al., 2016).

### Bacillus subtilis

Mutation in *ykcB*, a glycosyltransferase believed to use C55-P-glucose for glycosylating cell surface components, resulted in decreased vancomycin susceptibility in *B. subtilis*. This mutation also affected the bacteria’s resistance to several antibiotics, making them more sensitive to *β*-lactams (Ishikawa et al., 2022).

### Burkholderia multivorans

Collateral sensitivity to multiple drugs such as levofloxascin, minocycline, chloramphenicol, and trimethoprim-sulfamethoxazole has been found in β-lactam meropenem-evolved strains of *B. multivorans*. Additionally, the role of an altered cell wall has been reported as a possible mechanism for this particular collateral sensitivity. Meropenem targets cell wall synthesis, and the altered cell wall may also enhance the accumulation of other drugs inside the cells (Flanagan et al., 2020).

Drug resistance *B. multivorans* isolated from the patients with cystic fibrosis who received either sulfamethoxazole-trimethoprim or meropenem treatment showed sensitivity to five antibiotic classes. Resistance to sulfamethoxazole-trimethoprim showed CS to meropenem, ceftazidime, levofloxacin, minocycline, and chloramphenicol. Whereas, resistance to recognized mutations involving regulator of putative resistance-nodulation-division efflux pump or other pumps can contribute to this altered sensitivity in *B. multivorans* (Kavanaugh et al., 2021).

In another study, collateral sensitivities were evident in 170 out of 279 evolved *B. multivorans* strains, with at least 25% showcasing reciprocal pairs of CS antibiotics. Notably, the pairs with the maximum reciprocal CS frequency, include meropenem – trimethoprim-sulfamethoxazole, meropenem - levofloxacin, meropenem - minocycline, ceftazidime - trimethoprim-sulfamethoxazole, and ceftazidime - levofloxacin (Kavanaugh et al., 2020). This study identified some of the candidate genes associated with the promising pairs of reciprocal sensitivity pairs. Levofloxacin-adapted isolates, exhibiting heightened susceptibility to meropenem, developed distinctive mutations in *rseP, dacB*, and *fimV*. Conversely, meropenem-adapted isolates, displaying increased susceptibility to levofloxacin, developed mutations in *rimO*, polyketide cyclase similar to *snoaL*, and *rhaT* (Kavanaugh et al., 2020). Levofloxacin-adapted isolates, exhibiting heightened susceptibility to ceftazidime, developed distinct mutations in *bpeR, fimV*, phage lysozyme. Conversely, ceftazidime-adapted isolates, displaying increased susceptibility to levofloxacin, developed distinctive mutations in *rne*, *dacB*, tRNA-ser, *fabI*, and a transmembrane protein (Kavanaugh et al., 2020).

### Enterococcus faecalis

In *E. faecalis*, a noteworthy and consistently observed CS to rifampicin emerged when mutants were selected using inhibitors of cell wall synthesis, such as ampicillin, oxacillin, ceftriaxone, and fosfomycin. This intriguing phenomenon suggests a potential relationship between the mechanisms involved in cell wall synthesis inhibition and the heightened susceptibility to RIF. Additionally, the study revealed a prevalent occurrence of cross-resistance to daptomycin (DAP) when cells were selected using other frequently used antibiotics, highlighting complex interactions and potential shared resistance mechanisms in the antibiotic response of *E. faecalis* (Maltas and Wood, 2019).

Another research study found significant variations in collateral responses to different antibiotics, particularly Ceftriaxone. Cross-resistance appeared in mutants harboring daptomycin-resistance mutations in cardiolipin synthetase (*cls*) or genes associated with the YxdJK two-component signaling system (*bceR* or *ycvR*). In contrast, mutations in *liaX*, part of the LiaFSR two-component system, are linked to ceftriaxone sensitivity. These findings uncover unique phenotypic variations in mutations affecting the LiaSFR system, emphasizing the trade-offs between daptomycin resistance and collateral responses, particularly to ceftriaxone (Huynh et al., 2023).

### Enterococcus faecium

The rise in sensitivity to lefamulin, a pleuromutilin antibiotic that targets the bacterial ribosome, is linked to vancomycin resistance in *E. faecium*. Epistasis among the *van* gene cluster and *msrC*, producing an ABC-F protein that guards bacterial ribosomes from the action of antibiotics, mediates the trade-off between the action of vancomycin and pleuromutilins (Li et al., 2022). Pleuromutilin medication is more effective than conventional therapy in reducing colonization and improving survival in of vancomycin-resistant *E. faecium* infection mice models (Li et al., 2022).

### Escherichia coli

Two regulatory systems (PmrB-PmrA and PhoP-PhoQ) control aminoglycoside (e.g., Gentamycin (Roemhild et al., 2020)) resistance mediated by *pmrB* in a systemic way. This leads to the modification of lipid A in the outer membrane, which lowers the membrane’s negative charge, which in turn increases aminoglycoside-resistant *E. coli*’s sensitivity to *β*-lactam antibiotics (Lázár et al., 2013; Baym et al., 2016).

CS against nitrofurantoin (NIT) is observed in *E. coli* and *S. enterica* mutants resistant to β-lactam antibiotic mecillinam, antimicrobial peptide protamine, and tigecycline (Roemhild et al., 2020). The observed heightened hypersensitivity in mutants (*hemL* and *lon*) and partial hypersensitivity in the *spoT* mutant can be elucidated by upregulation of nitroreductase activity, resulting in overexpression of oxygen-insensitive nitroreductases NfsA and/or NfsB (Roemhild et al., 2020). The *hemL* mutant displayed heightened antibiotic uptake rates, indicating a common mechanism of collateral sensitivity involving increased active intracellular antibiotic concentrations (Roemhild et al., 2020).

**Figure 1:**
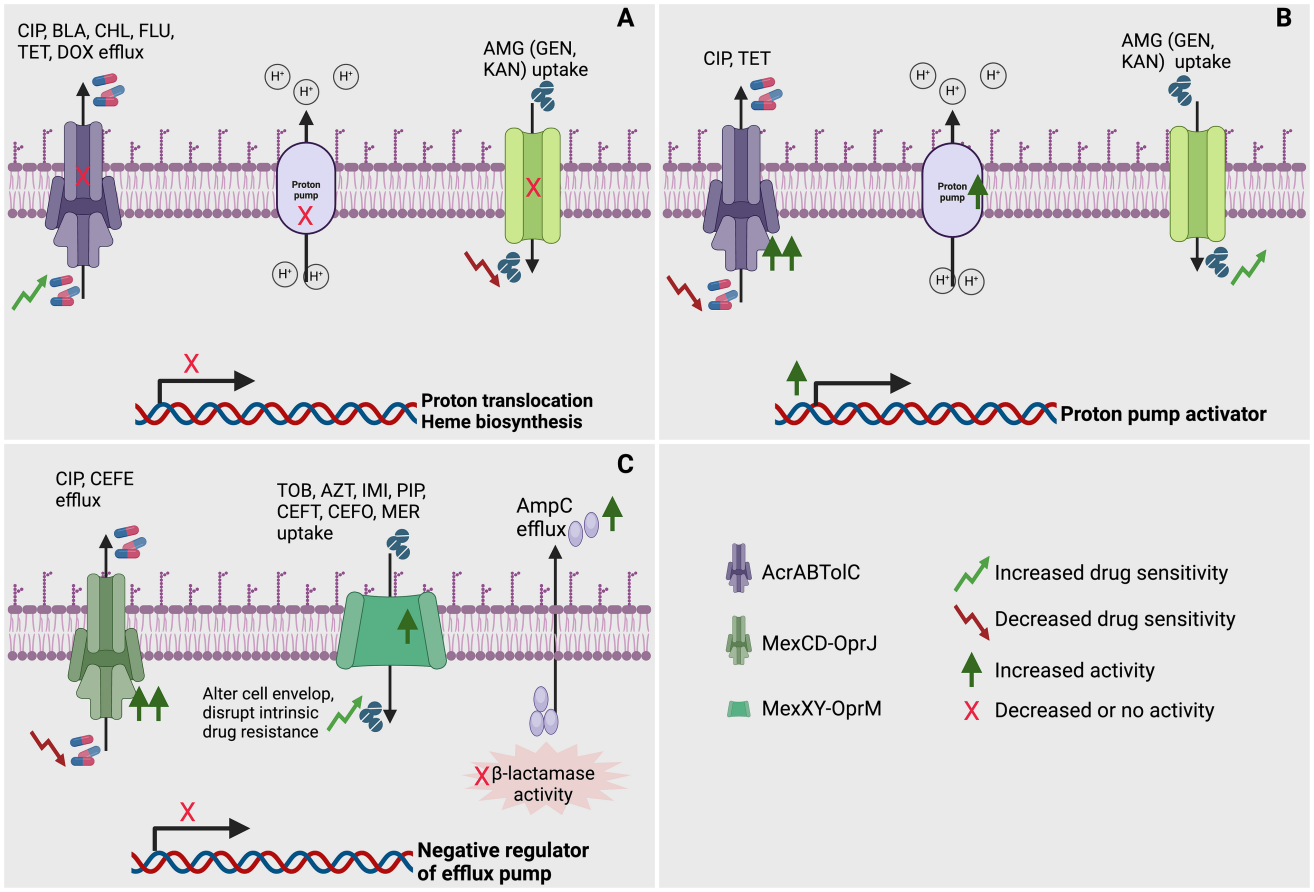
Role of efflux pump in achieving collateral sensitivity (CS) in bacteria. **(A)** Mutations affecting genes involved in proton translocation or heme biosynthesis might impair the production of a proton motive force, potentially hindering the expulsion of CIP, CEF, BLA, CHL, FLU, TET, and DOX via H^+^-powered efflux pumps. Additionally, these mutations could also impede the uptake of specific AMG (GEN and KAN) through alternative efflux pumps (Lázár et al., 2013; Hasan et al., 2023). **(B)** Upregulation of genes such as proton pump activators enhance proton translocation, potentially facilitating the expulsion of CIP and TET via efflux pumps. Additionally, these could also facilitate the uptake of specific AMG (GEN and KAN) through alternative efflux pumps (Hasan et al., 2023). **(C)** Mutations impacting the negative regulator of efflux pump genes could modify the cell envelope and disturb inherent drug resistance, potentially amplifying the efflux of specific CIP and CEFE via efflux pumps. Simultaneously, they could augment the expulsion of certain TOB, AZT, IMI, PIP, CEFT, CEFO, and MER by upregulating specific efflux pumps. Furthermore, functional mutations might diminish beta-lactamase activity by increasing efflux (Hernando-Amado et al., 2023a; Yen and Papin, 2017; Mulet et al., 2011). In this figure, CIP, ciprofloxacin; CEF; BLA, β-lactams; CHL, chloramphenicol; FLU, fluoroquinolones; TET, tetracycline; DOX, doxycycline; GEN, gentamycin; KAN, kanamycin; CIP, ciprofloxacin; TOB, tobramycin; AZT, aztreonam; IMI, imipenem; PIP, piperacillin; CEFT, ceftazidime; CEFE, cefepime; CEFO, cefotaxime; MER, meropenem; AcrABTolC, resistance-nodulation-division (RND) efflux pump; MexCD-OprJ, resistance-nodulation-division (RND) efflux pump; MexXY-OprM, resistance-nodulation-division (RND) efflux pump. Created using BioRender.

In a different investigation, the heightened sensitivity to *β*-lactam antibiotics observed in aminoglycoside-resistant *trkH* mutants is attributed to a decrease in the activity of the multidrug efflux pump AcrAB-TolC. This reduction in pump activity results from a diminished membrane potential in *E. coli* (Lázár et al., 2013). Furthermore, hypersensitivity to additional antibiotics (beta-lactams, chloramphenicol, fluoroquinolones, tetracycline, and doxycycline) may arise from *E. coli*’s resistance to aminoglycoside as a consequence of reduced PMF and PMF dependent efflux-pump through mutation in *TrkH, CyoB, IspA* and *HemA* (Lázár et al., 2013; Figure 1A).

Horizontal gene transfer is a crucial factor in the dissemination of antibiotic resistance. This study proposes that carbapenem-resistant plasmid pOXA-48–carrying *E. coli* can be eradicated by using azithromycin and colistin based on the evolved CS (Herencias et al., 2021). Another study explored CS in the extended spectrum *β*-lactamase CTX-M-15 mutant of *E. coli*. Identifying mutations on horizontally transmitted β-lactamase gene blaCTX-M-15, that increase resistance to mecillinam or piperacillin–tazobactam combination may increase susceptibility to cephalosporin drug cefotaxime. *In vitro* and in mice, a mecillinam and cefotaxime combination effectively eradicated both wild-type and resistant CTX-M-15 (Rosenkilde et al., 2019).

The induction of the AcrAB-TolC efflux pump, resulting from a mutation and subsequent decline in the *marR* gene in *E. coli*, reduces susceptibility to multiple antibiotics such as ampicillin, cefoxitin, tetracycline etc. Alternatively, the mutation in *marA* increases the expression of the kinase *WaaY*, which may phosphorylate the inner core of lipopolysaccharides. This process enhances the negative charge of the outer membrane of bacteria and, consequently, increases susceptibility to cationic antimicrobial peptides such as glycine-leucine-amide, human beta defensin-3, etc. (Lázár et al., 2018).

Furthermore, a potent collateral sensitivity in borrelidin A analogs against cephalosporin-resistant *E. coli* was identified, which is linked to the known target, tRNA ligase (ThrRS), particularly in strains with cell wall biosynthesis mutations (Liu et al., 2023).

### Klebsiella pneumoniae

Tetracyclines and aminoglycosides showed reciprocal CS with carbapenem-resistant *K. pneumoniae*. While the imbalanced oxidation–reduction process of strains resistant to tetracycline may cause an increase in bacterial sensitivity to antibiotics like aminoglycosides, the greater sensitivity of aminoglycoside-resistant strains to tetracyclines was linked to changes in bacterial membrane potential (Ma et al., 2023). Interestingly, increased reactive oxygen species levels are linked to the activation of the efflux pump SoxRS-MarAB-AcrAB (Zhao and Drlica, 2014). In tetracycline-resistant strains, we also found a notable elevation of the oxidoreductase activity and oxidation–reduction process, which may disrupt the homeostasis in bacteria and enhance the antibacterial effect of aminoglycosides (Ma et al., 2023; Belenky et al., 2015; Van Acker and Coenye, 2017).

Carbapenem-resistant *K. pneumoniae* may show CR to eravacycline, through the elevated expression of multidrug efflux pump AcrA-AcrB-TolC, as a consequence of mutation in the *Lon* protease gene (Xu et al., 2022). Furthermore, due to the overexpression of the porin proteins OmpA and OmpU, the developed resistant mutants exhibit CS to the *β*-lactam / β-lactamase inhibitor combinations ceftazidime-avibactam and aztreonam/avibactam (Xu et al., 2022). Crucially, in a mouse cutaneous abscess model, eravacycline plus either ceftazidime-avibactam or aztreonam/avibactam showed synergistic therapeutic benefits (Xu et al., 2022).

An enzyme compromise occurs when the structure of *K. pneumoniae* carbapenemase is altered in a way that makes it resistant to ceftazidime-avibactam. At the same time, decreased enzymatic activity makes them collaterally sensitive to imipenem, meropenem, and ertapenem sensitivity (Hobson et al., 2022).

### Mycobacterium tuberculosis

Historically, intrinsic β-lactam resistance in *M. tuberculosis* restricted their use in tuberculosis treatment. Recent reports of sensitivity in resistant strains selected for classical tuberculosis therapy (isoniazid, rifampicin, streptomycin, amikacin, levofloxacin, ethambutol, ofloxacin, pyrazinamide, and ethionamide) renewed interest, but the molecular understanding lagged. The activation of the major inhibitor of *β*-lactam resistance, *blaI*, and associated transcriptional connections between genes regulate vulnerability and resistance, hinting at regulatory explanations for collateral sensitivity to β-lactams (Trigos et al., 2021). Furthermore, cross-resistance between bedaquiline and clofazimine is caused by the upregulation of efflux pump Mmlp15 due to the mutation in the transcriptional regulator rv0678 (Hartkoorn et al., 2014).

INH-resistant mutant of *M. tuberculosis* may show increased CS to bedaquiline (BDQ), TB47, PA824, TAC, and Q203 because of defective *katG* in the resistant mutant strain (Waller et al., 2023). Conversely, various *rpoB* (i.e., RIF and FIX), *atpE* (i.e., BDQ), and *katG* (i.e., INH) mutations with their adapted antibiotic showed greater sensitivity to PA824, besides multiple strains showed higher sensitivity to Q203 a cytochrome bc1:aa3 inhibitor (Waller et al., 2023).

### Pseudomonas aeruginosa

Under normal growth conditions of *P. aeruginosa*, the expression of efflux pump MexCD-OprJ encoding genes remains low. However, isolates resistant to antibiotic exhibit an overproduction of this efflux pump are selected in the presence of diverse antibiotics. This overproduction is attributed to the mutation acquisition resulting in loss of function in the gene *nfxB*, known as its negative regulator (Figure 1C). For example, in *P. aeruginosa* PAO1, the amplification of the MexCD-OprJ efflux pump, triggered by the *nfxB* mutation, has proven to be a substantial factor in elevating resistance to ciprofloxacin (Hernando-Amado et al., 2023a; Yen and Papin, 2017; Mulet et al., 2011) and cefepime (Mulet et al., 2011) (Figure 1C). Surprisingly, this heightened efflux pump activity renders the bacterium susceptible to a spectrum of other antipseudomonal agents. Particularly noteworthy is the increased vulnerability to imipenem (Mulet et al., 2011), piperacillin (Yen and Papin, 2017), and tobramycin (Hernando-Amado et al., 2023a; Yen and Papin, 2017; Mulet et al., 2011) (Figure 1C). Moreover, a heightened sensitivity is evident across various other *β*-lactams, encompassing ceftazidime, cefotaxime, piperacillin-tazobactam, aztreonam, and meropenem, as demonstrated through comprehensive experimental testing (Mulet et al., 2011). This overexpression of MexCD-OprJ induces significant alterations in the cell envelope physiology of *P. aeruginosa*. As a consequence, it disrupts the foundational elements of the bacterium’s intrinsic resistance, affecting key components such as the major constitutive efflux pumps (MexAB-OprM) and inducible efflux pumps (MexXY-OprM), as well as the inducible AmpC-lactamase (Figure 1C) (Mulet et al., 2011). This investigation further highlighted that the observed phenotypes are directly linked to the overexpression of the efflux pump rather than the inactivation of its regulatory NfxB. Additionally, it disrupted a crucial mutation-driven mechanism for *β*-lactam resistance, namely constitutive AmpC overexpression. This disruption was marked by a substantial reduction in periplasmic β-lactamase activity, seemingly caused by an abnormal efflux of AmpC out of the bacterial cell (Mulet et al., 2011). Moreover, altered drug uptake and efflux ratios explain collateral sensitivity in ciprofloxacin-resistant *nfxB* mutants of *P. aeruginosa*, with consistent aminoglycoside sensitivity and, notably, sensitivity to colistin in clinical isolates evolved to resist azithromycin and ciprofloxacin (Imamovic et al., 2018).

In another study, varying sequence types and distinct preexisting mutational resistomes were identified in *P. aeruginosa* strains that were resistant to ciprofloxacin. Despite having these genetic variations, the study emphasized the consistent collateral sensitivity to tobramycin and aztreonam, suggesting a potential application of this evolutionary insight for eliminating *P. aeruginosa* infections completely (Hernando-Amado et al., 2023b).

Examining collateral effects, another study conducted the experimental evolution of 160 isolates of *P. aeruginosa* to achieve high resistance levels against eight frequently used antibiotics. This research brought to light varied patterns of CS and CR within populations that had adapted to identical antibiotics. Genomic and functional genetic analyses demonstrated that mutations in various regulatory genes, such as *mexZ* or, *nalC*, played a role in aminoglycoside sensitivity in isolates that achieved *β*-lactam resistance. Furthermore, mutations in the two-component regulatory system gene *pmrB* were associated with heightened penicillin (piperacillin-tazobactam, carbenicillin) sensitivity in populations that had developed resistance to gentamicin (Barbosa et al., 2017). Similarly, in cystic fibrosis patients exposed to antibiotic treatment, *P. aeruginosa* strains that are resistant to gentamicin have been observed to develop sensitivity to penicillin, attributed to a mutation in a two-component system (*pmrB*). Additionally, *β*-lactam-adapted strains with mutations in *nalC* and *mexZ* exhibit sensitivity to aminoglycosides (Jansen et al., 2016).

A robust CS pattern to fosfomycin was found in antibiotic-resistant mutants of *P. aeruginosa*, which were specifically selected using antibiotics from different structural families, such as tobramycin, tigecycline, or ceftazidime. Fosfomycin gains entry into the cell through the GlpT transporter, and once inside, it can be inactivated by FosA. The decreased expression of *fosA* results in an increased intracellular concentration of functional fosfomycin. Functional fosfomycin, in turn, inhibits *de novo* peptidoglycan synthesis by targeting MurA. Under these conditions, the synthesis of peptidoglycan may be sustained by the peptidoglycan-recycling pathway. Disruption of this pathway, due to compromised function, leads to heightened sensitivity to fosfomycin (Laborda et al., 2022; Genova et al., 2023).

Another study investigated the potential of exploiting a trade-off observed in pyomelanogenic-resistant mutants of *P. aeruginosa* coexisting with other phenotypic variants in cystic fibrosis patients. The results indicated that it is possible to eliminate pyomelanogenic mutants by initially using tobramycin, followed by guiding the remaining population to develop increased susceptibility to tobramycin through the use of ceftazidime that, in turn, selects ceftazidime resistance. The chromosomal deletion of the *mexXY* gene that encodes the multidrug efflux pump may be responsible for this trade-off. It’s noteworthy that populations obtained after tobramycin/ceftazidime alternation also displayed CS to Fosfomycin, which can further eliminate the evolved resistance population (Hernando-Amado et al., 2020).

### Salmonella enterica

CS to the Cyclotide Cycloviolacin O2, an antimicrobial peptide, was observed in *S. enterica* through mutations in *pmrA*, related to lipopolysaccharide biosynthesis and selected for colistin resistance, as well as a *hemL* mutation selected for resistance to protamine. In contrast, cross-resistance to antimicrobial peptides and/or antibiotics distinct from the ones they were initially selected for was noted due to several occurrences of mutations in *sbmA, phoP, hemL*, and *pmrA* (Malik et al., 2017).

### Staphylococcus aureus

One research study examined extracts from six Vietnamese medicinal plants for their antibacterial effects on *S. aureus* and its other variants. Through *in vitro* adaptation to the extracts over 30 passages under sub-lethal concentrations, *S. aureus* gradually adapted to the extracts while swiftly developing resistance to antibiotics (Nguyen et al., 2022). Strains adapted to plant extracts showed collateral sensitivity to antibiotics (streptomycin, chloramphenicol, kanamycin). Whereas, strains adapted to antibiotics exhibited CR to both antibiotics and extracts, emphasizing *S. aureus*’s low resistance to antimicrobial plant extracts (Nguyen et al., 2022).

In addition, vancomycin and/or daptomycin adapted *S. aureus* showed more susceptibility to β-lactam antibiotics. *S. aureus* also showed increased susceptibility to ceftaroline once they became resistant to vancomycin, daptomycin, and teicoplanin (Barber et al., 2014). Clinical studies have shown that using daptomycin in conjunction with β-lactams effectively prevents and treats infections caused by daptomycin-resistant MRSA strains. VraSR plays a critical role in daptomycin resistance by leading to mutations in *mprF*, potentially affecting PrsA chaperone function needed for the posttranscriptional maturation of penicillin-binding protein 2a. These findings suggest how daptomycin-resistant strains may regain sensitivity to β-lactams targeting cell wall components (Renzoni et al., 2017; Jiang et al., 2022). Daptomycin-resistant *S. aureus* often has gain-in-function mutations in the *mprF* gene, which helps maintain positive surface charge. Standard β-lactams, despite being less effective against MRSA, may prevent these mutations and revert daptomycin-resistant isolates to daptomycin-susceptible phenotypes. This study found that β-lactams targeting penicillin-binding protein −1 significantly increased daptomycin susceptibility in daptomycin-resistance isolates. Prolonged β-lactam exposure can lead to additional *mprF* mutations that may alter the cell envelope and metabolism, additionally enhancing susceptibility to host defense peptides and reducing surface charge (Jenson et al., 2020).

In another study, cross-resistance to glycopeptides, lipopeptides, and lipoglycopeptides has been observed among daptomycin, vancomycin, and dalbavancin-adapted MRSA strains (Hines et al., 2020). In addition, a seesaw effect against various β-lactams, depending on the specific penicillin-binding protein targets, has also been observed in MRSA strains. Membrane composition modifications were found in daptomycin, vancomycin, and dalbavancin-adapted strains. Interestingly, increased phosphatidylglycerols were found to be associated with increased resistance to glycopeptides, lipopeptides, and lipoglycopeptides and increased susceptibility to β-lactams (Hines et al., 2020). A different study revealed an *in vitro* “seesaw” effect between daptomycin and oxacillin in MRSA, where the development of resistance to daptomycin was accompanied by a reduction in resistance to oxacillin (Yang et al., 2010). In an *in vitro* PK/PD model, ceftaroline and vancomycin were tested against isogenic MRSA strains with varying susceptibilities to vancomycin, showing that ceftaroline was more effective against mutant strains with increased vancomycin resistance (Werth et al., 2013).

### Staphylococcus haemolysticus

An interesting seesaw effect was observed in daptomycin-resistant *S. haemolyticus*. Daptomycin-adapted *S. haemolyticus* becomes more susceptible to penicillin despite the presence of the *bla* operon and b-lactamase activity. Similarly, susceptibility to cefoxitin increased notably, even though the *mecA* gene and *mec* operon were still detected (Vignaroli et al., 2011).

### Salmonella typhimurium

*Salmonella typhimurium* adapted to gentamicin and kanamycin may exhibit cross-resistance to these antibiotics and CS to tetracycline and ciprofloxacin. This outcome is attributed to the diminished activity of AcrAB-TolC caused by the dissipation of protonmotive force (PMF) (Hasan et al., 2023; Figure 1A). Tetracycline-adapted *S. typhimurium* exhibited cross-resistance to ciprofloxacin and vice versa. The study also revealed CS in tetracycline and ciprofloxacin-adapted *S. typhimurium* to gentamicin and kanamycin, linked to thein AcrAB-TolC efflux pump (Figure 1B) and OmpC porin proteins (Hasan et al., 2023). The AcrAB-TolC efflux pump utilizes strong PMF to expel antibiotics and other toxins, thereby conferring resistance to fluoroquinolones while potentially increasing susceptibility to aminoglycosides (Hasan et al., 2023).

### Phage

Different polysaccharides, including capsular polysaccharides, exopolysaccharide antigens, and lipopolysaccharides on bacterial membranes, may serve as phage receptors. In contrast, the alteration of these polysaccharides may make the bacteria resistant to phage (Fujiki et al., 2023; Mangalea and Duerkop, 2020; Uchiyama et al., 2016; Bertozzi Silva et al., 2016). Interestingly, altered polysaccharides may increase collateral sensitivity to antibiotics or antimicrobial peptides in phage-resistant strains (Fujiki et al., 2023).

Beyond the polysaccharides mentioned earlier, membrane proteins such as efflux pumps can also function as phage receptors (Mangalea and Duerkop, 2020; Bertozzi Silva et al., 2016; Ofir and Sorek, 2018). Drug efflux capability is expected to diminish in response to phage resistance-induced mutation or deletion of the drug efflux pump, hence increasing antibiotic sensitivity (Fujiki et al., 2023). Using phages that kill harmful bacteria as well as facilitate the evolution of a phage-resistant population that are then susceptible to conventional treatments is a useful tactic known as “phage steering.”

In *Burkholderia cenocepacia*, complete lipopolysaccharide and membranes confer serum resistance. Bacterial susceptibility to serum immune components and membrane-targeting antibiotics such as colistin is increased by phage-induced structural alterations in LPS (Ruest et al., 2023).

## Challenges and opportunities

### Limitations and challenges in studying collateral sensitivity

**Figure 2:**
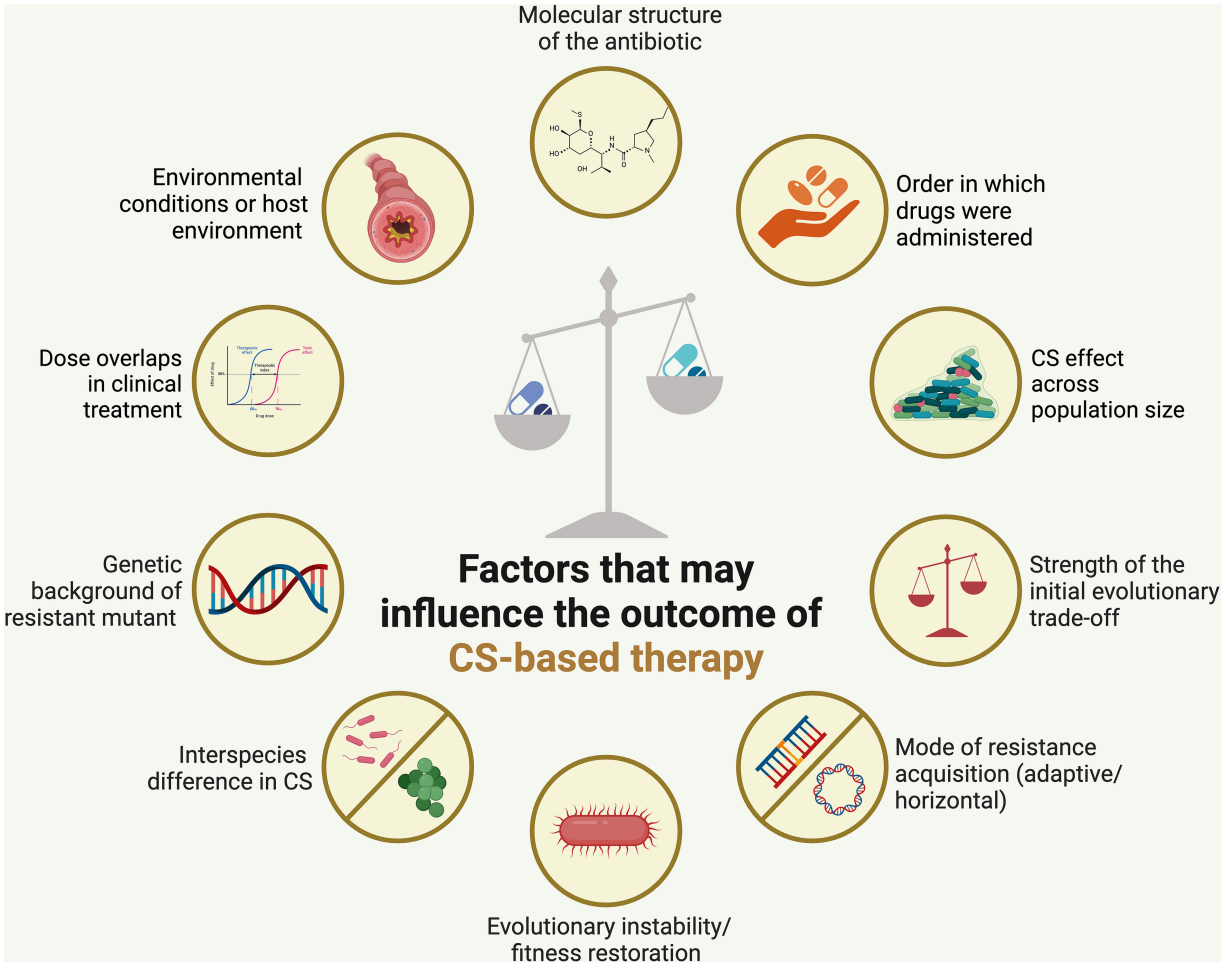
Factors affecting the stability of collateral sensitivity (CS)-based treatment. Employing CS-based antibiotic therapy could help mitigate the emergence of antibiotic resistance, although the effectiveness of this approach is heavily influenced by several key factors. Created using BioRender.

CS has garnered significant attention recently as a strategy to curb the rise of drug-resistant pathogens and enhance treatment outcomes. It holds potential as a treatment approach to counter the swift evolution of antibiotic resistance globally. However, various factors must be carefully considered to establish it as a promising therapeutic approach (Figure 2). These factors are discussed below.

### Evolutionary instability/fitness restoration

Evolutionary instability in collateral networks, such as compensatory mutation related to newly acquired resistance determinants, may weaken the premise of effective CS therapeutic strategies. For example, a study on clinical *E. coli* strains resistant to ciprofloxacin found that following 300 generations of growth without antibiotic treatment, strains with four out of the five genetic backgrounds regain their fitness completely (Sørum et al., 2022). This study demonstrated that initial collateral networks could be undermined by decreased efflux pump expression through compensatory mutations. It identified *rpoS* as a potential target for compensatory evolution (Sørum et al., 2022). Therefore, understanding the role of compensatory evolution in restoring bacterial fitness is a crucial factor to succeed in CS-based therapy.

### Different genetic background

Despite being adapted to the same drug, distinct patterns of CS and CR may arise; concurrently, the bacterial genetic background may influence the effectiveness of CS (Hernando-Amado et al., 2023a; Barbosa et al., 2017; Genova et al., 2023). The patterns stem from mutation stochasticity and varied evolutionary tracks to resistance against the initial therapy, resulting in particular collateral effects on other therapeutic drugs (Barbosa et al., 2017; Nichol et al., 2019). The strength of this hypersusceptibility trait among bacteria with different genomic backgrounds—which are frequent in long-term human infections—presents a hurdle when implementing it (López-Causapé et al., 2018). When exposed to a second medication, separate populations selected with the same treatment should dependably show comparable collateral effects; nevertheless, this high repeatability is not found constantly (Barbosa et al., 2019).

### Interspecies difference in CS

CS depends unquestionably on how much similar species’ resistance mechanisms and trade-offs coincide (Pál et al., 2015). A study examined collateral effects in 160 independent populations of *P. aeruginosa*, rapidly evolving resistance to eight antibiotics. It revealed both CS and CR, with patterns differing from those reported in other bacterial species, implying interspecific variations in evolutionary trade-offs exists and should be considered in designing rational therapy (Barbosa et al., 2017).

### Horizontal gene transfer

Mobile genetic elements—particularly plasmids—are essential for the spread of antimicrobial resistance (ABR) genes among clinical pathogens and are a primary cause of the concerning global increase in ABR (Partridge et al., 2018). Adaptive evolution has historically been studied extensively to identify CS interactions for chromosomal mutations. However, CS for horizontally acquired resistance genes has not been thoroughly investigated, nor has the effectiveness of drug combinations been thoroughly examined (Li et al., 2022; Munck et al., 2014; Gonzales et al., 2015; Imamovic et al., 2018; Wang et al., 2023). Therefore, it would not be possible to gain a complete understanding of CS in bacteria without paying similar attention to plasmid-mediated CS in bacteria.

### Selecting model organisms to study laboratory CS

While much research concentrates on finding patterns of CS in model strains, given that infections often involve bacteria already resistant to antibiotics, robustness across varied genetic backgrounds, including already drug-resistant isolates, is necessary for practical applicability (Hernando-Amado et al., 2023b; Zwep et al., 2021; Lieberman et al., 2014). Fortunately, the robustness of CS may also allow for therapeutic application despite genetic differences. Notably, CS to aztreonam and tobramycin showed promise for perhaps eliminating *P. aeruginosa* infections (Hernando-Amado et al., 2023b).

### Environmental conditions or host environment

Evaluating the robustness of collateral effects under a range of environmental variables is crucial for lab-to-clinic translation. The expression of collateral effects varies for individual mutations and favors different sets of mutants with distinct collateral sensitivities, depending on the local circumstances (pH, temperature, and bile) (Allen et al., 2021). For instance, zinc can decrease the activity of enzymes that break down aminoglycosides, bile can increase efflux pumps, and in the absence of antibiotics, high temperatures can alter how rifampicin resistance mutations affect (Rosenberg et al., 2003; Lin et al., 2014; Rodríguez-Verdugo et al., 2013). CS interactions, on a broader scale, could be influenced by the local environmental conditions that bacteria encounter. This variability might diminish repeatability and pose challenges to clinical application (Allen et al., 2021).

### Population size

The evolution of CS varies, and it should ideally be consistent across conditions. When it comes to an antibiotic combination, small populations exhibit constant CS, whereas bigger populations exhibit complete cross-resistance. This difference is most likely caused by a higher likelihood of advantageous rare mutations (Barbosa et al., 2019; Jiao et al., 2016).

### Dose overlaps

Variations arise between the controlled laboratory environment (*in vitro*) and the dynamic context within a living organism (*in vivo*). Notably, the swift manipulation of drugs in the lab is not replicated in the body. Interestingly, medications can be changed quickly in the lab, but patients’ antibiotic concentrations change because of pharmacokinetic processes, which include dose overlaps from successive administrations. Through interactions between drugs, these overlap phases can affect the evolutionary dynamics (Nyhoegen and Uecker, 2023).

### Emerging opportunities and potential applications

CS is less advantageous in the absence of pre-existing resistance unless cell division is sluggish. For treatment to be effective, rapid drug cycling that takes into account drug–drug interactions is essential. Fast-cycling antibiotics may be able to stop the emergence of resistance, according to lab research, which makes collaterally sensitive medications a good fit for these kinds of treatments (Nyhoegen and Uecker, 2023). Furthermore, addressing knowledge gaps may be possible with the growing availability of clinical antibiotic sensitivity data, especially Minimum Inhibitory Concentration (MIC) values. Recent research has shown that discrete resistance values can be used to estimate collateral effects; nevertheless, in order to evaluate the therapeutic relevance of CS, more comprehensive clinical MIC data must be analyzed in order to infer directionality and impact magnitude (Zwep et al., 2021). Recognizing the polymicrobial nature of infections, it is essential to identify the MIC in polymicrobial conditions, which can be accomplished through a polymicrobial checkerboard assay (Black et al., 2023). It is also crucial to consider the inhibitory activity of an antibiotic combination in polymicrobial conditions when designing CS-based therapy. This is because a combination that proves effective in monoculture may not necessarily yield the same effectiveness in a polymicrobial community. For instance, the combination of gentamicin and ceftazidime exhibits synergy against *P. aeruginosa* in monoculture but shows antagonism in a polymicrobial culture (Black et al., 2023).

Characterizing recently acquired resistance determinants in relation to a susceptible wild type in an ideal setting is usually how collateral networks are identified in research. The prediction of these networks depends on the evolutionary stability of the first connection between resistance determinants and hosts of bacteria, highlighting the need to take this into account when developing treatment plans (Sørum et al., 2022). The order in which drugs are administered is a critical factor in identifying the potential vulnerability that bacteria may develop when using combination therapy that focuses on CS. As a result, medication pairings can be ranked according to the likelihood of developing collateral resistance as long as the model takes into account the variety of resistance mutations that the bacteria can acquire (Ardell and Kryazhimskiy, 2021).

## Future directions in research

It has been observed that successful utilization of evolved CS in successive treatment is dependent on treatment order and combination, CS impact magnitude and directionality, fitness costs of resistance, adaptive trade-offs, and epistatic genetic linkages (Barbosa et al., 2019; Aulin et al., 2021).

There are still questions about the best times to use CS-based dosing, the effects of drug–drug interactions, and how to schedule treatments optimally. In order to resolve these uncertainties, future research should concentrate on developing customized multi-drug antibiotic dosage schedules and improving CS-based therapy techniques (Aulin et al., 2021; Nyhoegen and Uecker, 2023). In an effort to close the gap between laboratory and clinical settings, mathematical modeling could be a promising approach to look at drug- and pathogen-specific aspects of various treatment plans in order to address contemporary issues (Aulin et al., 2021). Furthermore, the mathematical model can be used to illustrate the range of collateral effects in bacteria treated with a particular medication. Their method correctly estimated the chance that a population will eventually develop CS or CR to a second antibiotic (Ardell and Kryazhimskiy, 2021; Torella et al., 2010). To this end, several other approaches, such as flux balance analysis, a computational models (Krueger et al., 2016), or fitness landscape analysis of resistance evolution, (Szendro et al., 2013) could be promising to develop rational CS-based therapy.

Collateral effects are essential in countering antibiotic resistance, but detecting them in clinical surveillance data is lacking. One study proposed a methodology by employing a conditional t-test, to assess MIC values for 419 *E. coli* strains and 20 tested antibiotics (Zwep et al., 2021). This approach, available as an R package, systematically identifies collateral effects, guiding future combination therapy and prescribing strategies in large-scale population surveillance studies.

Its potential as a therapeutic focus is predicated on the idea that the exploited trade-off is hard to overcome and has evolved to be stable. Because of this, it ought to either eradicate bacterial populations or reduce the development of multidrug resistance by causing resensitization to one of the antibiotics (Barbosa et al., 2019). Consequently, directing further research efforts toward the identification of the most common resistant mutations for a specific pathogenic organism and antibiotic is imperative. This targeted approach can enhance our understanding of resistance mechanisms and contribute to the development of more effective therapeutic strategies. Moreover, expanding the scope of research to identify similar resistant mutations in other bacteria can broaden the applicability of findings. By exploring shared resistance mechanisms across bacterial species, we may uncover common vulnerabilities and potential targets for intervention. This approach not only enriches our knowledge of antibiotic resistance but also holds promise for the development of broadly applicable solutions to combat resistance in diverse bacterial populations.

A popular method for evaluating bacteria’s CS and CR is to use the minimum inhibitory concentration (MIC). However, there is a lack of research on how these variations in susceptibility relate to the antibiotic concentration known as the mutant prevention concentration (MPC), which stops a single-step mutation (Lozano-Huntelman et al., 2020).

Studying acquired drug resistance through horizontal gene transfer is crucial in CS-based therapy, where random mutagenesis could prove to be a valuable tool to explore resistance evolution. Furthermore, random mutagenesis’s ability to investigate CS patterns is helpful in that it offers a practical means of simulating evolutionary processes, makes large-scale antibiotic screenings easier, and gets around some of the drawbacks of using conventional adaptive laboratory evolution techniques to study individuals horizontally transferred genes (Pál et al., 2015; Andersson and Hughes, 2010).

## Clinical implications and applications

The success observed in these different treatment modalities signifies a significant step forward in the ongoing battle against drug resistance, showcasing the effectiveness of collateral CS as a promising avenue for developing innovative and more sustainable solutions in the field of drug-resistant reemergence. For example, strong CS to tobramycin and aztreonam is produced when ciprofloxacin generates clinically significant resistance mutations in *P. aeruginosa* mutants that already exist. When combining ciprofloxacin and aztreonam instead of alternating between the two drugs, antibiotic-resistant mutants are more effectively driven to extinction (Hernando-Amado et al., 2022). Clinical implementation demands stable, repeatable collateral effects of resistance development (Sørum et al., 2022). Ensuring the persistence of CS across bacterial strains with varying mutational backgrounds is indispensable for its effective translation into clinical practice. It is especially important to investigate the resilience of CS in previously established antibiotic-resistant mutants with exposure to a novel antimicrobial; nevertheless, this situation has not been extensively studied in a clinical context (Hernando-Amado et al., 2022).

Mutual CS requires physiological justification and experimental validation prior to clinical application. The goal of suggested clinical approaches, such as antibiotic cycling and combination therapy, is to prevent the emergence of resistance in common infections at the individual and institutional levels (Udekwu and Weiss, 2018). Antibiotic sequencing is critical to the effectiveness of CS-based cycling treatments, particularly, antibiotic kind, CS directionality, and subpopulations of resistance should be considered to provide a personalized infectious disease treatment (Waller et al., 2023; Aulin et al., 2021). Notably, for antibiotics with a limited therapeutic window, CS-based therapies exhibit the greatest promise (Aulin et al., 2021). Furthermore, it has been found that treatment failure is unavoidable when antibiotic concentrations are low because resistant mutants emerge quickly. Treatment effectiveness is improved, and resistance is prevented by using multiday cycling in conjunction with moderate to high dosages of particular bacteriostatic and bactericidal antibiotic combinations (Udekwu and Weiss, 2018).

## Conclusion

CS relationships between antibiotics can be unidirectional, where reduced sensitivity to one antibiotic induces collateral sensitivity to a second antibiotic but not vice versa, or reciprocal, where reduced sensitivity to either of the antibiotics leads to collateral sensitivity to the other (Aulin et al., 2021). Reciprocal collateral sensitivity is considered a crucial factor for effective treatments based on collateral sensitivity, although these relationships are less frequently observed compared to one-directional scenarios (Imamovic and Sommer, 2013; Podnecky et al., 2018; Gonzales et al., 2015).

Typically, alterations in bacterial physiology, such as modifications in cell membrane permeability or the expression of drug targets, have the potential to affect the phenomenon of CS to antibiotics (Ma et al., 2023). Due to regulatory changes, the altered lipopolysaccharide constituents on the outer membrane of bacteria has role in CS in drug resistant bacteria (Lázár et al., 2018). Typically, the expression of genes encoding efflux pumps is low during regular growth conditions. Nevertheless, antibiotic-resistant isolates, characterized by an upregulation of efflux pumps resulting from mutations in the regulatory genes, are frequently isolated from patients undergoing treatment. This increased expression, stemming from regulatory gene mutations, may contribute to the facilitation of CS (Hernando-Amado et al., 2023a). For a combination of two drugs to be effective, certain criteria need to be fulfilled. One key consideration is the belief that optimal killing efficiency and therapeutic selectivity are achieved when the combined effect of the drugs is synergistic, surpassing the sum of their individual effects (Lehár et al., 2009).

Overall, to design a rational trade-off or CS of therapy, the following factors should be considered (Figure 1). These include (i) the structure of the antibiotic to which bacteria developed sensitivity, (ii) the order in which drugs were administered, (iii) whether resistance incurred a substantial fitness cost (i.e., when resistance-promoting genetic alterations hinder the bacteria’s normal replication and survival), and (iv) the strength of the initial evolutionary trade-off (i.e., the extent of bacteria’s sensitivity) (Barbosa et al., 2019), (v) mode of collateral sensitivity acquisition (through adaptive evolution or horizontal gene transfer) (Li et al., 2022; Munck et al., 2014; Gonzales et al., 2015; Imamovic et al., 2018; Wang et al., 2023), (vi) chance of evolutionary instability/fitness restoration (Sørum et al., 2022), (vii) interspecies difference in CS (Barbosa et al., 2017), (viii) versatility in the genetic background of resistant mutant (Hernando-Amado et al., 2023a; Barbosa et al., 2017; Genova et al., 2023), (ix) types of model organisms to study laboratory CS (Hernando-Amado et al., 2023b), (x) environmental conditions or host environment (Allen et al., 2021), (xi) CS effect across population size (Barbosa et al., 2019; Jiao et al., 2016), and (xii) dose overlaps in clinical treatment (Nyhoegen and Uecker, 2023).
